# Dynamics of symbiotic bacterial community in whole life stage of *Harmonia axyridis* (Coleoptera: Coccinellidae)

**DOI:** 10.3389/fmicb.2022.1050329

**Published:** 2022-12-01

**Authors:** Lingen Du, Hui Xue, Fangmei Hu, Xiangzhen Zhu, Li Wang, Kaixin Zhang, Dongyang Li, Jichao Ji, Lin Niu, Junyu Luo, Jinjie Cui, Xueke Gao

**Affiliations:** ^1^State Key Laboratory of Cotton Biology, Institute of Cotton Research, Chinese Academy of Agricultural Sciences, Anyang, China; ^2^Zhengzhou Research Base, State Key Laboratory of Cotton Biology, School of Agricultural Sciences, Zhengzhou University, Zhengzhou, China; ^3^Hubei Insect Resources Utilization and Sustainable Pest Management Key Laboratory, Huazhong Agricultural University, Wuhan, China

**Keywords:** *Harmonia axyridis*, symbiotic bacteria, high-throughput sequencing, quantitative PCR, life stages

## Abstract

**Introduction:**

Bacteria play critical roles in the reproduction, metabolism, physiology, and detoxification of their insect hosts. The ladybird beetle (*Harmonia axyridis*) harbors a myriad of endosymbiotic microbes. However, to date, little is known about how the microbial composition of *H. axyridis* varies throughout its life cycle.

**Methods:**

In this study, 16S rRNA amplicon sequencing and quantitative PCR were employed to investigate the diversity and dynamics of bacterial symbionts across the egg, larval, pupae, and adults stages of *H. axyridis*.

**Results:**

Higher bacterial community richness and diversity were observed in eggs, followed by those in adults and pupae. The community richness index differed significantly between second-instar larvae and other developmental stages. Proteobacteria, Firmicutes, and Actinobacteria were the dominant phyla. *Staphylococcus, Enterobacter, Glutamicibacter*, and *Acinetobacter* were the dominant bacteria genera; however, their relative abundances fluctuated across host developmental stages. Interestingly, the larval stage harbored high proportions of Firmicutes, whereas the adult microbial community largely consisted of Proteobacteria.

**Discussion:**

This study is the first to determine the symbiotic bacterial composition across key life stages of *H. axyridis*. These outcomes can foster the development of environmental risk assessments and novel biological control strategies.

## Introduction

The existence of symbiotic relationships between insect hosts and their internal microbiota is a broad and widely recognized phenomenon (Douglas, 2014). Microbial endosymbionts play important roles in host insects, contributing to nutrient acquisition, development and reproduction, detoxification and metabolism, and strengthening the immune system against pathogen and parasitoid invasion (Douglas, 1998; Dillon and Dillon, 2004; Koch and Schmid-Hempel, 2011; Shin et al., 2011; Zheng et al., 2013). Examples of such contributions include the synthesis of vitamins and amino acids, detoxification of toxic compounds, stimulation of the immune system and defense against pathogens, protection against natural enemies, and facilitation of tolerance against abiotic stressors (Scarborough et al., 2005; Jones et al., 2013; Gerardo and Parker, 2014; Douglas, 2015; Kwong et al., 2017; Muhammad et al., 2017; Horak et al., 2020). Studies have shown that symbiotic bacteria in the genus *Buchnera* promote aphids’ fitness by providing essential amino acids (Douglas et al., 2001). Additionally, *Citrobacter,* a gut endosymbiotic bacterium of *Bactrocera dorsalis,* enhanced resistance against the organophosphate insecticide trichlorfon (Cheng et al., 2017). However, the microbiota of many insect species have not yet been well characterized, making it difficult to understand the composition and function of bacterial symbionts and their potential impacts on host ecology and evolution.

*Harmonia axyridis* is an important natural enemy in cotton fields. It is used as a biological control agent in agricultural and forest systems, owing to its high fertility and strong predation capacity (Kenis et al., 2020), and has been employed successfully as a biological control agent in many countries (Brown et al., 2008). Both larval and adult stages are polyphagous and prey upon various destructive agricultural pests (Muhammad et al., 2017), which play an important role in natural and agricultural ecosystems (He et al., 2018). Additionally, *H. axyridis* is a useful model species for environmental risk assessments (Desneux et al., 2007; Gao et al., 2021).

*Harmonia axyridis* is holometabolous, with four gradual stages (egg, larva, pupa, and adult) proceeding development. Currently little is known about the bacterial community structures across life stages, and how microbial populations change as a function of metamorphosis. Therefore, investigating the composition of bacterial endosymbionts across all life stages of *H. axyridis* is necessary. Moreover, it is important to understand the bacterial species richness, composition dynamics, and functional organization of endosymbionts for future studies on microbial symbiosis in this important insect species (Verstraete, 2007; Marzorati et al., 2010).

In this study, the bacterial community composition and relative abundance of eggs, larvae, pupae, and adults (male and female) of *H. axyridis* were investigated *via* high-throughput Illumina sequencing of the bacterial 16S rRNA gene fragment. Additionally, we investigated how *H. axyridis*-associated bacterial species richness, composition dynamics, and relative abundances varied by life stage, as well as the absolute abundance. Other studies have shown that differences in bacterial relative abundances determined by high-throughput techniques may not accurately reflect those of actual taxon abundances (Zhang et al., 2017). Therefore, we used 16S rRNA gene-targeted group-specific primers for quantitative PCR analysis of four identified predominant bacterial genera across the different developmental stages of *H. axyridis*. Detailed elucidation of *H. axyridis* bacterial symbionts will not only provide comprehensive insights into understanding the composition and functional roles of bacterial endosymbionts, but also contribute to the development of biological control strategies.

## Materials and methods

### Rearing and collection of *Harmonia axyridis*

*Harmonia axyridis* used in this study were collected from the field of Institute of Cotton Research of CAAS (Anyang, Henan, China). The *H. axyridis* population were reared in cages (35 cm × 35 cm × 35 cm) under the following conditions: 25°C ± 2°C; 14: 10 h light: dark cycle; and 70 ± 10% relative humidity (RH). Beetles were fed on pea aphids, *Acyrthosiphon pisum*, reared on *Vicia faba* plants in an artificial climate chamber under the following conditions: 20°C ± 2°C, 14: 10 h light: dark cycle, and 70% ± 10% relative humidity (RH).

All samples were collected within 24 h of emergence, including eggs, first-, second-, third-, and fourth-instar larvae, pupae, female adults, and male adults, and flash frozen in liquid nitrogen. One hundred individual eggs were pooled as a single sample; six repeats were used for each instar, with 20 individuals each instar pooled as a single sample.

### DNA extraction and PCR amplification

Prior to DNA extraction, all samples were surface sterilized by washing 70% ethanol for 30 s, soaking in H_2_O_2_, followed by rinsing thrice with sterile water. TIANamp Genomic DNA Kit (TIANGEN Biotech, Beijing, China) was used for microbial DNA extraction. DNA concentration and purity were quantified using the NanoDrop 2000C spectrophotometer (Thermo Scientific, United States), and DNA quality was checked by 1% agarose gel electrophoresis.

For the bacterial 16S rRNA gene PCR amplification, the V3-V4 region of the 16S rRNA gene was amplified using the primer set 338F 5-ACTCCTACGGGAGGCAGCAG-3 and 806R 5-GGACTACHVGGGTWTCTAAT-3 (Chu et al., 2015) using a GeneAmp 9,700 thermocycler PCR system (ABI, United States). PCR reactions were carried out in triplicate in 20 μl mixtures that included 10 ng of template DNA, 4 μl of 5 × FastPfu Buffer, 2 μl of 2.5 mM dNTPs, 0.4 μl of FastPfu Polymerase, 0.8 μl (5 μM) of each primer, and nuclease-free water. The cycling conditions were as follows: initial denaturation at 95°C for 3 min, 29 cycles of 95°C for 30 s, 53°C for 30 s, and 72°C for 45 s, and a final extension at 72°C for 10 min. PCR products were visualized on 2% agarose gels after amplification.

### Illumina MiSeq platform sequencing

PCR products were excised from 2% agarose gels, purified using the AxyPrep DNA Gel Extraction Kit (Axygen Biosciences, Union City, CA, USA), and quantified using QuantiFluor™-ST (Promega, United States) according to the manufacturer’s protocol. The purified amplicons were pooled in equimolar amounts, and sequencing was conducted on an Illumina MiSeq PE300 platform by Majorbio Co., Ltd. (Shanghai, China). A flowchart of the experimental procedure is provided in Supplementary Figure S1.

### Bioinformatic analysis of 16S rDNA and statistical methods

Raw sequence reads of all samples were demultiplexed, quality-filtered using Trimmomatic, and merged using FLASH (Mago and Salzberg, 2011). The optimized sequences were clustered into operational taxonomic units (OTUs) using UPARSE (version 7.1, http://drive5.com/uparse/) based on sequence similarity of 97% (Edgar, 2013). Subsequently, all effective 16S rRNA gene sequences were aligned against the SILVA (SSU132) 16S rRNA database.^1^ Then, through the Ribosomal Database Project (RDP, http://rdp.Cme.msu.edu/) Classifier (Quast et al., 2012), the species classification information corresponding to each OTU was obtained using a 70% confidence threshold.

Based on the OTU results, rarefaction curves and alpha diversity indices referring to community diversity (Shannon and Simpson), community richness (Chao and Ace), and sequencing depth (Good’s coverage) were estimated using MOTHUR^2^ (Schloss et al., 2009; Caporaso et al., 2010). The differences in alpha diversity among groups were compared using one-way ANOVA for normally distributed data; non-normally distributed samples were subjected to the Kruskal–Wallis test to compare between treatments. A heatmap was generated from the relative abundance of OTUs using the Vegan Package in R (version 2.4; https://cran.r-project.org/web/packages/vegan/). For phylogenetic beta diversities, non-metric multidimensional scaling analysis (NMDS) was used for visualization using community membership and the relatedness of community members. To detect potential biomarkers, the linear discriminant analysis (LDA) effect size (LEfSe) method^3^ was used based on a normalized relative abundance matrix. The LEfSe method uses the Kruskal–Wallis test to identify features with significant differences across all life stages of *H. axyridis* and performs LDA to evaluate the effect size of each feature (Segata et al., 2011). In addition, Phylogenetic Investigation of Communities by Reconstruction of Unobserved States (PICRUSt) was used to predict the Kyoto Encyclopedia of Genes and Genomes (KEGG) category and obtain the levels of metabolic pathway information (Langille et al., 2013).

### Quantification of bacterial communities

Quantitative PCR (qPCR) was used to determine total copies of the 16S rRNA gene using universal bacterial 16S rDNA primers 27F (5′-AGAGTTTGATCCTGGCTCAG-3′) and 355R (5′-CTGCTGCCTCCCGTAGGAGT-3′) on a StepOnePlus™ Real-Time PCR System (Applied Biosystems, Foster City, CA, United States). 20 μl reactions of 10 μl 2× TransStart Green qPCR SuperMix (TransGen Biotech, China), 1 μl of template DNA, 0.4 μl (each) 10 μM primer, 0.4 μl 50 × ROX, and 7.8 μl Nuclease-free-water, were performed in triplicate. The cycling conditions were 95°C for 3 min followed by 40 cycles of 95°C for 5 s and 60°C for 30 s. Quantification was based on standard curves produced from serial dilutions of the cloned target sequence in the pEASY-T3 cloning vector (TransGen Biotech, China). Each reaction plate had an independent standard curve. Additionally, we also determined the copy number of the eight most dominant bacterial genera following the same PCR conditions described above; primers and PCR efficiencies are shown in Supplementary Table S2.

### Sequence data accession number

All 16S rRNA gene sequence data are available in the Sequence Read Archive under accession no. SRP254074.

## Results

### Overview of *Harmonia axyridis* microbiotas

In this study, we performed Illumina MiSeq sequencing of the bacterial 16S rRNA gene to analyze. After quality filtering and read merging, a total of 2,731,854 high-quality reads remained for subsequent analysis, which clustered into 2,455 OTUs based on 97% sequence similarity (Supplementary Table S1). The highest and lowest OTUs number were observed in eggs and second-instar larvae, respectively.

**Figure 1:**
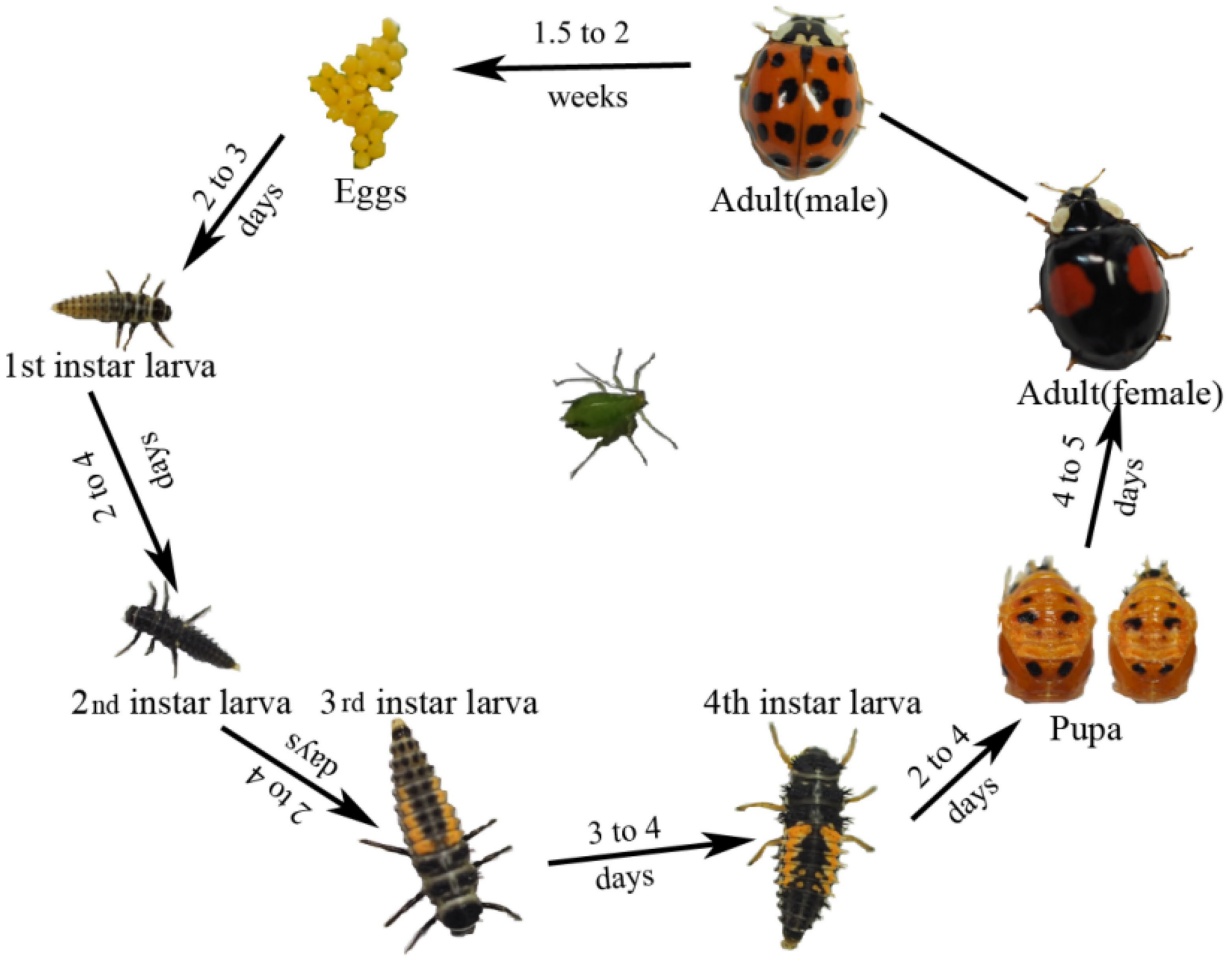
Overview of development stages of the *Harmonia axyridis*.

**Figure 2:**
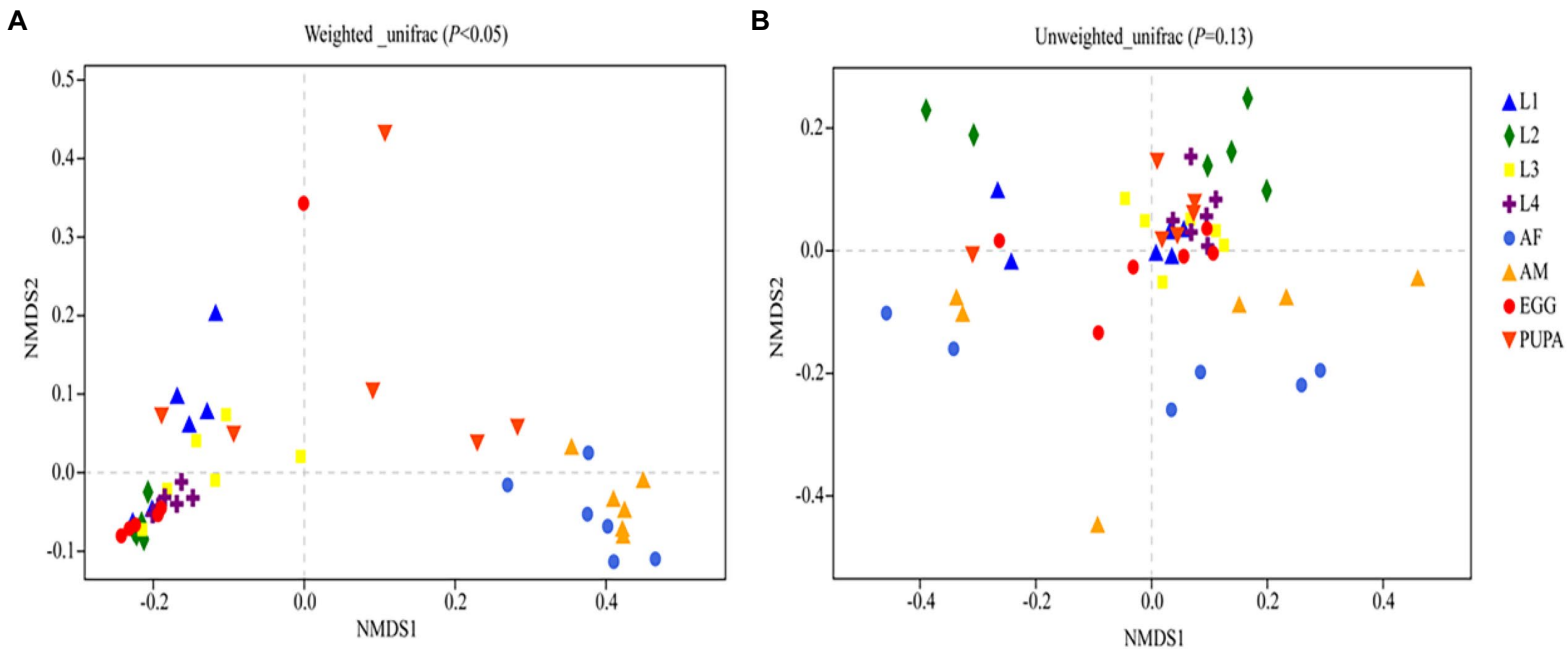
Non-metric Multidimensional Scaling (NMDS) ordination based on **(A)**, weighted and **(B)**, unweighted UniFrac distances of bacterial communities of *Harmonia axyridis*.

*Harmonia axyridis*, as a complete metamorphosis insect, has a complete life cycle including egg, larva, pupa, and adult (Figure 1). Alpha diversity indices were used to assess bacterial community richness (Chao1 and Ace) and diversity (Shannon and Simpson; Supplementary Figure S2; Supplementary Table S1). Significant differences in the community richness index were found between the second-instar larvae and all other stages (Supplementary Figure S2A), except for adults (Supplementary Figure S2B). The rarefaction curves of bacterial OTUs tended to be saturated (Supplementary Figure S3) and coverage values of all samples were more than 0.99 (Supplementary Table S1), suggesting that the sequencing depth and microbial diversity were enough to cover the majority of bacterial taxa present across all development stages of *H. axyridis*. We compared the community composition and structures of all samples using non-metric multidimensional scaling analysis (NMDS); the beta diversity of microbiota associated with different development stages of the ladybird was investigated by weighted and unweighted Unifrac. Diversity estimation suggested higher bacterial community richness and diversity in the eggs, followed by adults and pupae, with the second-instar larval stage having the least community richness and diversity (Figure 2).

### Microbial community composition

**Figure 3:**
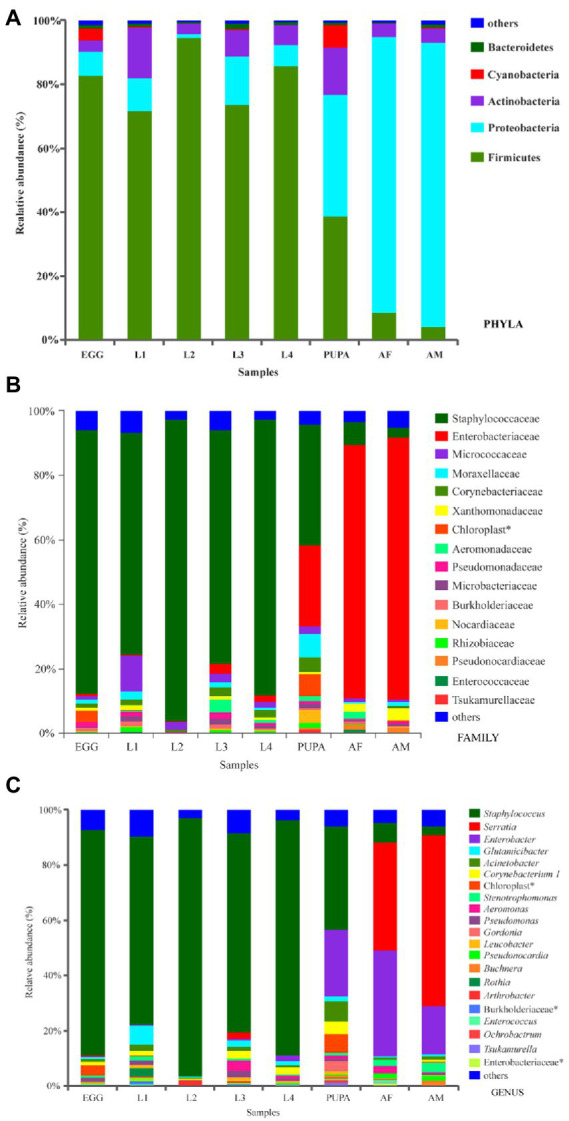
Bacterial community composition at the level of **(A)**, phyla (>0.01), **(B)**, family (>0.01) and **(C)**, genus (>0.01). *denotes unclassified operational taxonomic units (OTU) reported at higher taxonomic levels. Refer to Figure 1 for sample abbreviations.

Normalized sequences were aligned against the SILVA database and clustered into different taxonomic levels using a 70% threshold. A total of 36 phyla, 81 classes, 212 orders, 412 families, and 900 genera were identified (Figures 2, 3). Taxonomic classification revealed that Firmicutes was the most prevalent phylum in eggs and larval-stage samples, while Proteobacteria were the most prevalent in adult samples (both males and females) of *H. axyridis.* Figure 3A shows the microbial community composition at the phylum level (relative abundance > 0.1%); in all samples, the top four phyla with highest relative abundances were Firmicutes, Proteobacteria, Actinobacteria, and Cyanobacteria. The prevalence of Firmicutes was significantly higher in eggs (82.87%), first-instar larvae (71.70%), second-instar larvae (94.60%), third-instar larvae (73.76%), and fourth-instar larvae (85.97%), whereas it decreased sharply in the pupa (38.72%). After eclosion, the relative abundance of Firmicutes decreased (3.94%–8.58%) in adults, while the prevalence of Proteobacteria increased. Firmicutes (*F* = 33.39, df = 7, *p* < 0.001), Proteobacteria (*F* = 61.58 df = 7, *p* < 0.001), and Actinobacteria (*F* = 4.46 df = 7, *p* = 0.001) showed significantly different relative abundances across the entire life cycle.

Further comparison of the microbial communities was conducted at the family level; the community composition of bacterial families is presented in Figure 3B. A representative sample of 16 main families was screened, and 15 of these families were members of Firmicutes, Proteobacteria, and Actinobacteria. Within Firmicutes, Staphylococcaceae was the most abundant across all larval-stage samples, ranging widely from 69.05 to 93.75%; values ranged from 3.08 to 6.93% in adult samples. Comparatively, the most abundant taxa in the adult samples were Enterobacteriaceae, ranging narrowly from 78.99% to 81.36% (Figure 3B).

A community heatmap was generated to visualize the distribution of multiple OTUs across different developmental stages of *H. axyridis* (Supplementary Figure S4). Staphylococcus, Enterobacter, Glutamicibacter, Acinetobacter, Corynebacterium, Aeromonas, and Pseudomonas were the dominant genera (Supplementary Figure S4). The Staphylococcus genus was most abundant across the egg and all larval-stage samples, ranging widely from 68.30% to 93.75%, while it decreased sharply in the pupa (37.50%) and only accounted for 3.08%–6.93% in adult samples. In contrast, the Enterobacter genus was more abundant in adult samples, followed by the other dominant genera of Serratia (39.24%–62.22%), Glutamicibacter (0.66%–0.68%), and Acinetobacter (0.56%–1.37%). Additionally, it is noteworthy that Serratia was only found in adult and third-instar larval samples.

### Significant differences in microbial communities across different developmental stages

**Figure 4:**
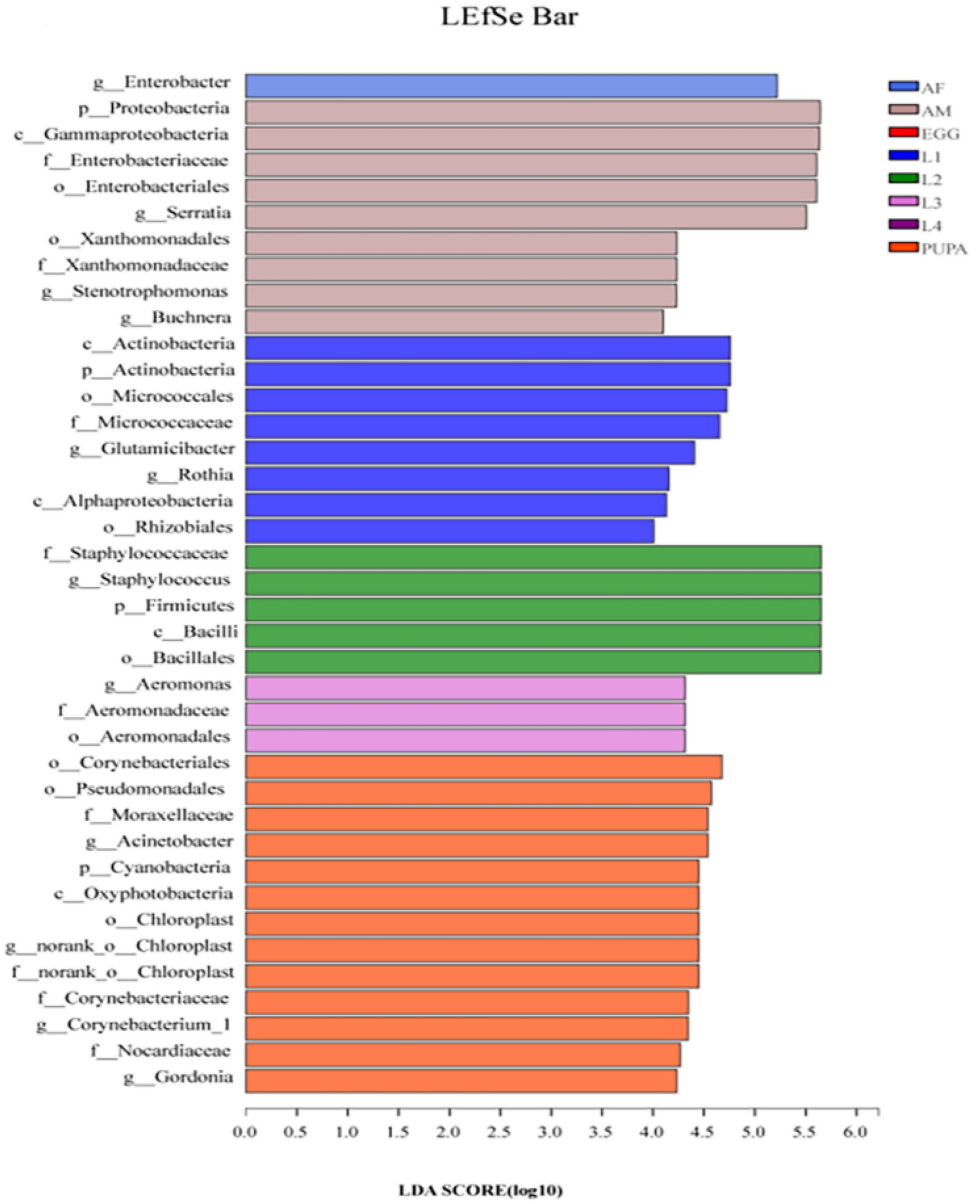
Linear discriminant analysis effect size (LEfSe) analysis of microbial abundance across all life stages of *Harmonia axyridis*. Linear discriminant analysis (LDA) score identified the size of differentiation across all life stages of *H. axyridis* with a threshold value of 4.0.

We performed biomarker analysis using the linear discriminant analysis (LDA) effect size (LEfSe) method to identify changes in the abundance of bacterial taxa associated with the observed differences across all life stages of *H. axyridis* (Figure 4). At the genus level, 11 bacterial clades presented statistically significant differences with an LDA threshold of 4.0 (Supplementary Figure S5). Among them, two bacterial genera were present in the first-instar larva, one in the second-instar larva, one in the third-instar larva, three in the pupa, one in female adults, and three in the male adults (Figure 4). The 11 genera showed significant differences in relative abundance across different developmental stages of *H. axyridis* (*p < 0.05*, non-parametric factorial Kruskal–Wallis (KW) sum-rank test). Combined with the heatmap of the relative abundance of bacterial genera (Figure 3) and community composition of bacterial genera (Figure 2B), the results demonstrated that five bacterial genera could be used as signatures of *H. axyridis* life stage. Specifically, the most representative bacterial genera in eggs and larval-stage samples were *Staphylococcus*, *Enterobacter*, *Glutamicibacter*, and *Acinetobacter*, and the most representative bacterial genera in adult samples were *Enterobacter*, *Serratia*, and *Staphylococcus*.

To verify the accuracy of culture-independent analysis of the dominant bacterial genera described above, we used 16S rRNA gene-targeted group-specific primers for real-time PCR analysis of four identified predominant bacterial genera (*Staphylococcus*, *Enterobacter*, *Glutamicibacter*, and *Acinetobacter*) and total bacterial abundance across the different developmental stages of *H. axyridis*. The total bacterial copy numbers at different developmental stages were significantly different (*p* < 0.005, Kruskal–Wallis test)—those of eggs and adults (females and males) were the highest, while those of second-instar larvae were the lowest. The copy numbers showed that larval stages had very high numbers of *Staphylococcus*, which harbored two dominant species, *Staphylococcus sciuri* and *Staphylococcus xylosus*. Uniquely, the bacterial genus *Acinetobacter* was found in higher abundance only in third-instar larvae, with copy numbers being 714.21-fold higher than in second-instar larvae, and 43.46-fold higher than in adults (Supplementary Figure S6). The bacterial community structure across life stages of *H. axyridis* was again validated using qPCR analysis, and was consistent with the previously described results of the microbial community.

### Functional prediction of microbiota

**Figure 5:**
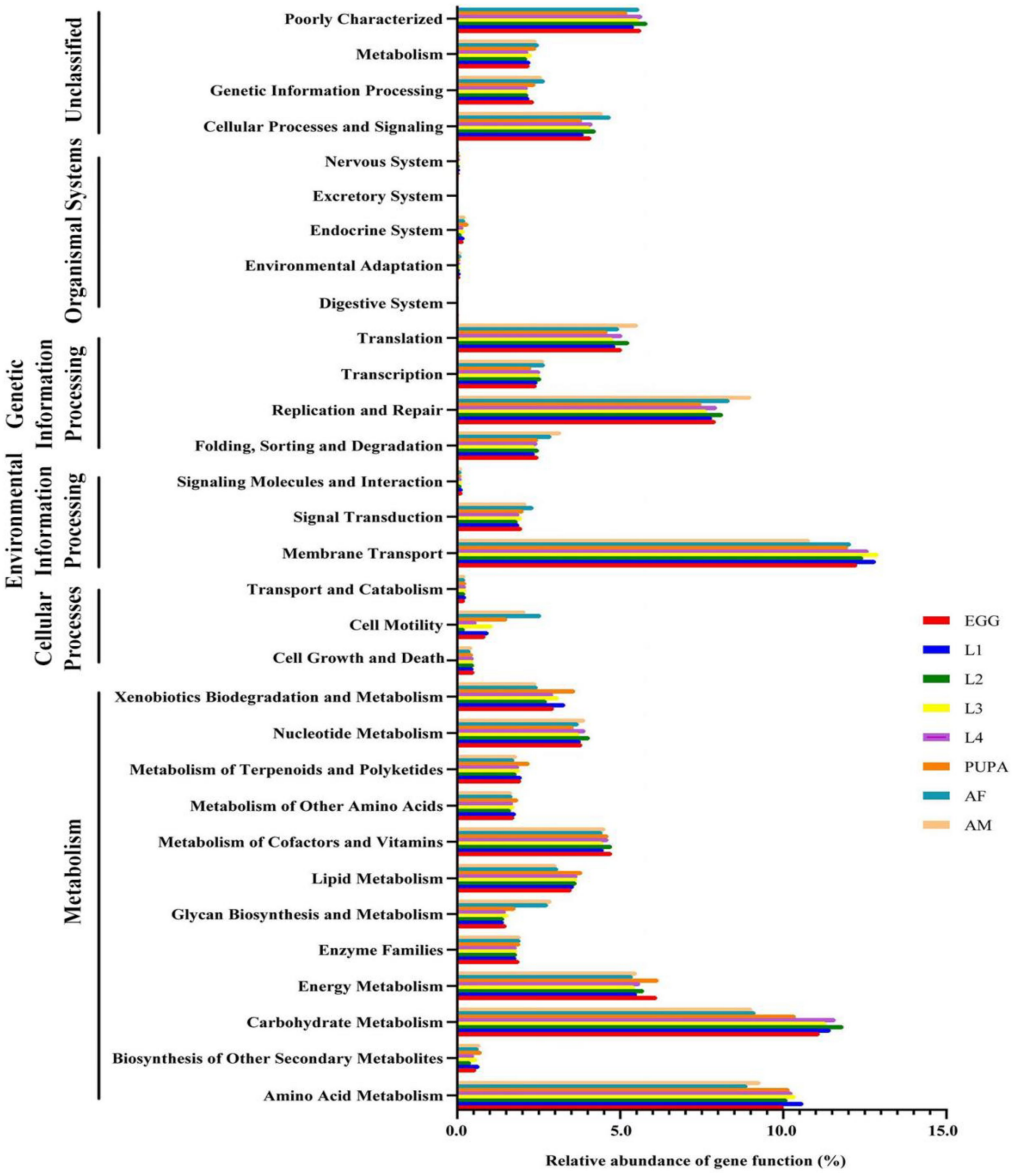
Inferred functions of bacterial communities associated with *Harmonia axyridis*. All of the predicted Kyoto Encyclopedia of Genes and Genomes (KEGG) metabolic pathways are shown at the second hierarchical level and grouped by major functional categories.

Using PICRUSt, we identified significant differences between the functional potentials of the bacterial community compositions across the different life stages of *H. axyridis*. We showed the pathway abundance at two levels: level 1 functional categories were displayed, including metabolism, environmental information processing, and genetic information processing at each developmental stage. In level 2, we showed the richness of the main pathways, including amino acid metabolism, carbohydrate metabolism, energy metabolism, and metabolism of cofactors and vitamins, presenting a major proportion of metabolic activity (Figure 5). On this basis, further dissection of the relationship between microbial abundance and functional roles is an important direction for further investigation.

## Discussion

Endosymbiotic bacteria of insects have recently become a key focus of much research because of their important roles such bacteria play in insect growth, development, reproduction, and host adaptability. Few studies have examined the microbial abundance and community structures of symbiotic bacteria across different developmental stages of insects, lacking comparisons of dynamic changes in microbial diversity (Eugeni et al., 2011; Salem et al., 2013). We used Illumina MiSeq deep sequencing of 16S rRNA genes to provide a comprehensive report of the bacterial communities in *H. axyridis* across all life stages.

*Harmonia axyridis* like other holometabolous insects, with each stage having distinct differences in morphology, structure, and function. The present study provides the first insight into the bacterial community structure, species richness, and dynamic changes across life stages (eggs, larvae, pupae, and adults (male and female)) of *H. axyridis*. This study found that a total of 2,455 OTUs were obtained from the sequencing results, and the annotated main taxonomic groups belonged to 36 phyla, 81 classes, 212 orders, 410 families, and 900 genera. Interestingly, diversity estimation analysis revealed that diversity indices and species richness varied across different developmental stages. Specifically, higher bacterial community richness and diversity were observed in eggs, followed by adults and pupae, and a significant difference was found in the community richness index between second-instar larvae and other stages. Overall, the results revealed that the diversity of bacterial communities differed significantly across different life stages; however, no effect was observed with respect to sex. Our study found that the microbial diversity of *H. axyridis* decreased significantly during the phase transition from egg to larva. This finding was similar to observations in other insects such as *Chrysoperla sinica* (Zhao et al., 2019), *Adelphocoris suturalis* (Xue et al., 2021), *Spodoptera exigua* (Gao et al., 2018), and *Chironomus* (Senderovich and Halpern, 2013). It is worth noting that the second-instar larvae stage of *H. axyridis* seems to be a special stage. We speculate that it may be the result of the interaction of species, food sources and living conditions, which needs further analysis and verification. Studies have shown that *Buchnera* is a primary symbiotic bacterium widely present in *A. pisum*. Secondary symbiotic bacteria in pea aphids in addition to the major *Serratia*, there are also *Wolbachia* and *Hamiltonella* (Martinez et al., 2014; Hrček et al., 2016; Skaljac et al., 2018). *Harmonia axyridis* began to prey on pea aphids in adult stage, which may explain the increase of *Serratia* composition in adult stage. Similar to previous reports on Drosophila, changes in the bacterial diversity of *H. axyridis* may be the result of a combination of diet (pea aphid feeding levels) and horizontal transmission (Chandler et al., 2011).

We also found that *H. axyridis* bacterial community were dominated by bacteria in the phyla Firmicutes and Proteobacteria, followed by Actinobacteria, Cyanobacteria, and Bacteroidetes across the entire life cycle (Figure 1A), which was similar to observations in other Coleoptera insects such as *Octodonta nipae* (Ali et al., 2019), *Propylea japonica* (Zhang et al., 2019), and *Anoplophora glabripennis* (Mason et al., 2018). The phylum Firmicutes frequently manipulates the bacterial community structure in various invertebrates, including *Deltocephalinae leafhoppers* (Kobia et al., 2018) and *Bactrocera dorsalis* (Huang et al., 2018). Firmicutes play significant roles in insect digestion, nutrient extraction, and organic matter degradation. Similarly, the phylum Proteobacteria can metabolize some secondary metabolites of insect host plants and maintain the development and reproduction of host insects (Dillon et al., 2002). Actinobacteria, another dominant group in *H. axyridis*, has been shown to play various metabolic and physiological functions including the synthesis of extracellular enzymes and secondary metabolites (Schrempf, 2001).

The most abundant genera in *H. axyridis* were *Staphylococcus*, *Enterobacter*, *Glutamicibacter*, *Acinetobacter*, and *Serratia*, unlike other ladybird species, among which Rickettsia, Wolbachia, and Spiroplasma bacteria were common (Weinert et al., 2007), but these observations were similar to those in *Propylea japonica* (Zhang et al., 2019). The Staphylococcus genus in phylum Firmicutes was dominant during the early developmental stages (eggs 81.94%, first-instar larvae 68.30%, second-instar larvae 93.75%, third-instar larvae 72.32%, fourth-instar larvae 85.46%, and pupae 37.50%). In nature, Staphylococcus infection is widespread and a large number of insects form symbiotic relationships with these bacteria. Unfortunately, the functional role of the genus *Staphylococcus* in insects is unclear. Adults had very high numbers of *Enterobacter*. *Enterobacter* has been reported to provide a number of substantial benefits to the biological traits of host insects, such as increased fecundity, shortened period of immature development stages, and improved male mating competitiveness and female mating receptivity (Kyritsis et al., 2019). This may be the best explanation for the enrichment of *Enterobacter* in the adult stage. *Serratia* is another dominant genus in the adult stage; in *Rhynchophorus ferrugineus*, *Serratia* has exhibited antimicrobial activity against bacterial pathogens and could be a potential biocontrol agent for pests (Scrascia et al., 2016). Throughout the development cycle of *H. axyridis*, *Glutamicibacter* and *Acinetobacter* were stable and continuous, with high abundances, indicating that they may play crucial roles in the growth and development of *H. axyridis*. *Glutamicibacter* and *Acinetobacter* are involved in many important functions, such as helping insects digest and absorb nutrients (Yang et al., 2014), providing protection against pathogens, and enhancing host fitness (Dillon and Dillon, 2004). Acinetobacter bacteria have strong drug resistance and play an important role in the immune response of insects because of their ability to degrade pesticides and other large molecular compounds, for their insect hosts (Hao et al., 2002; Li et al., 2020). These dominant symbiotic bacteria across all life stages of *H. axyridis* demonstrated important metabolic potential, suggesting that *H. axyridis* microbiota may play essential roles in their host physiology. Our analyses also revealed that metabolic processes were likely the main functional roles of microbial communities (Figure 3). In addition, the functions and pathways described above have so far only been based on the hypothesis found in other insects such as *P. japonica*, and may play different functions in the body of *H. axyridis*, which requires further testing and exploration.

Symbiotic bacteria can affect host fitness in many ways, including nutrition and regulating growth rate and stress tolerance (Ferrari et al., 2010; Ruokolainen et al., 2016). Although *H. axyridis* occupies a dominant position in field pest control, there is a lack of research on its bacterial diversity. We found that the dynamic changes of the flora of *Enterobacter* and *Serratia*, which are dominant from *Staphylococcus* to the final adult stage, are inseparable from the host development stage and dietary changes. The dominant genera of *H. axyridis* (*Staphylococcus*, *Enterobacter*, *Gluconobacter*, *Acinetobacter,* and *Serratia*) may play an important role in nutrient absorption, energy metabolism and environmental adaptation, thus affecting the development and reproduction of host insects. Finally, our study provides a better understanding of the diversity and community composition patterns of host-associated bacteria in different life cycle stages of ladybirds, which will provide an important theoretical basis for future research on symbiotic bacteria and help us understand the ecological and evolutionary roles of intestinal symbionts in this important insect assembly.

## Conclusion

As an important natural enemy, and a non-target surrogate in environmental risk assessment of Bt rice, the microbial structure of *H. axyridis* is of concern. This article investigated the diversity and dynamics of bacterial symbionts across the egg, larval, pupae, and adults stages of *H. axyridis.* We found that the symbionts structure of *H. axyridis* significantly varied with age development. In addition, we also predicted the biological functions of different bacterial communities, and provided a basis for further research on the role of bacteria in this and other insects. This is the first bacterial community report to address the life history of the beneficial insect in the field, *H. axyridis*. Our study reveals our understanding of the community structure of *H. axyridis* throughout its life cycle, and further advances the environmental risk assessment and biocontrol strategies of dominant natural enemies.

## Data availability statement

The datasets presented in this study can be found in online repositories. The names of the repository/repositories and accession number(s) can be found in the article/Supplementary material.

## Author contributions

JC, JL, and XG: conceptualization, software, and supervision. HX: methodology and visualization. LN and JJ: methodology and software. FH: investigation, data curation, and software. LD: writing and editing. KZ: data curation and formal analysis. XZ and LW: resources and investigation. DL: validation and investigation. All authors contributed to the article and approved the submitted version.

## Funding

This research was supported by Agricultural Science and Technology Innovation Program of Chinese Academy of Agricultural Sciences and China Agriculture Research System.

## Conflict of interest

The authors declare that the research was conducted in the absence of any commercial or financial relationships that could be construed as a potential conflict of interest.

## Publisher’s note

All claims expressed in this article are solely those of the authors and do not necessarily represent those of their affiliated organizations, or those of the publisher, the editors and the reviewers. Any product that may be evaluated in this article, or claim that may be made by its manufacturer, is not guaranteed or endorsed by the publisher.

## Footnotes

^1^ http://www.arb-silva.de

^2^ http://www.mothur.org/wiki

^3^ http://huttenhower.sph.harvard.edu/lefse/
