# Supplementary material for: Dynamics of symbiotic bacterial community in whole life stage of Harmonia axyridis (Coleoptera: Coccinellidae)
Source: Front Microbiol. 2022 Dec 1;13:1050329. doi: 10.3389/fmicb.2022.1050329 (PMC9751998; doi:10.3389/fmicb.2022.1050329)
Supplement: Supplementary file 1 [file Data_Sheet_1.docx]

**Supplementary Materials:**

Figure S1: The experimental procedure used for microbial community analysis in this study.

Figure S2. Rarefaction curves of the 16S rRNA gene reads based on OTUs at 97% sequence similarity.

Figure S3. Rarefaction curves of the 16S rRNA gene reads based on OTUs at 97% sequence similarity.

## Figure S4. Heat map of major taxa over across the *H. axyridis* life cycle, at the genus level.

Figure S5. Linear discriminant analysis effect size (LEfSe) analysis of microbial abundance across all life stages of *H. axyridis.*

Figure S6. Changes in gut microbiota composition across all life stages of *H. axyridis*.

**
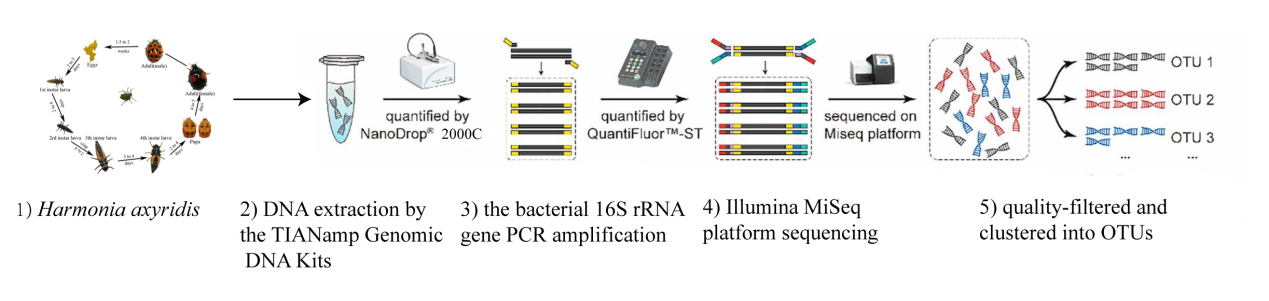
**

**Figure S1. The experimental procedure used for microbial community analysis in this study.**


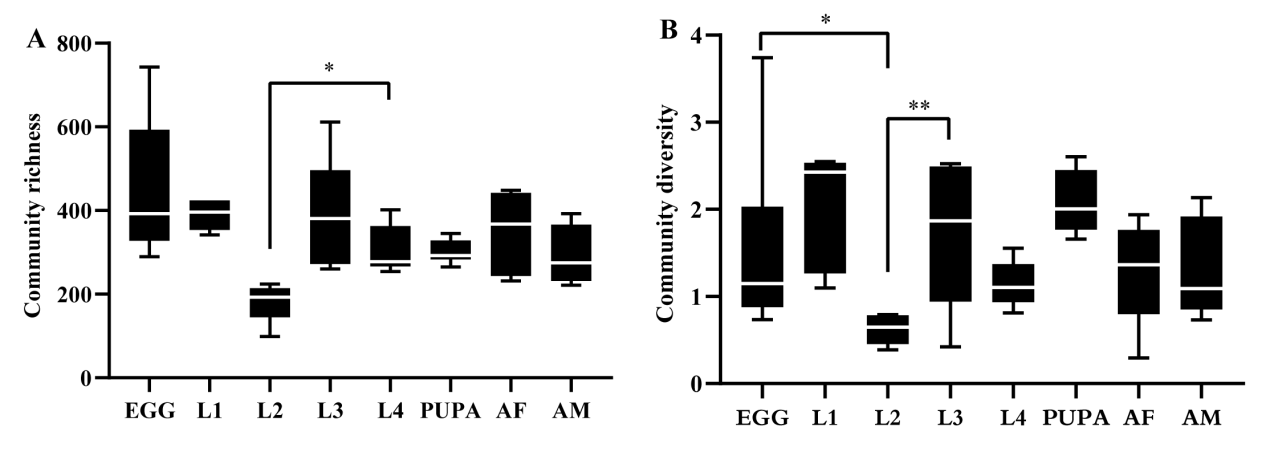


## Figure S2.Alpha diversity of all samples. (A) Community richness measured by the Chao index, (B) Community diversity measured by the Shannon index.


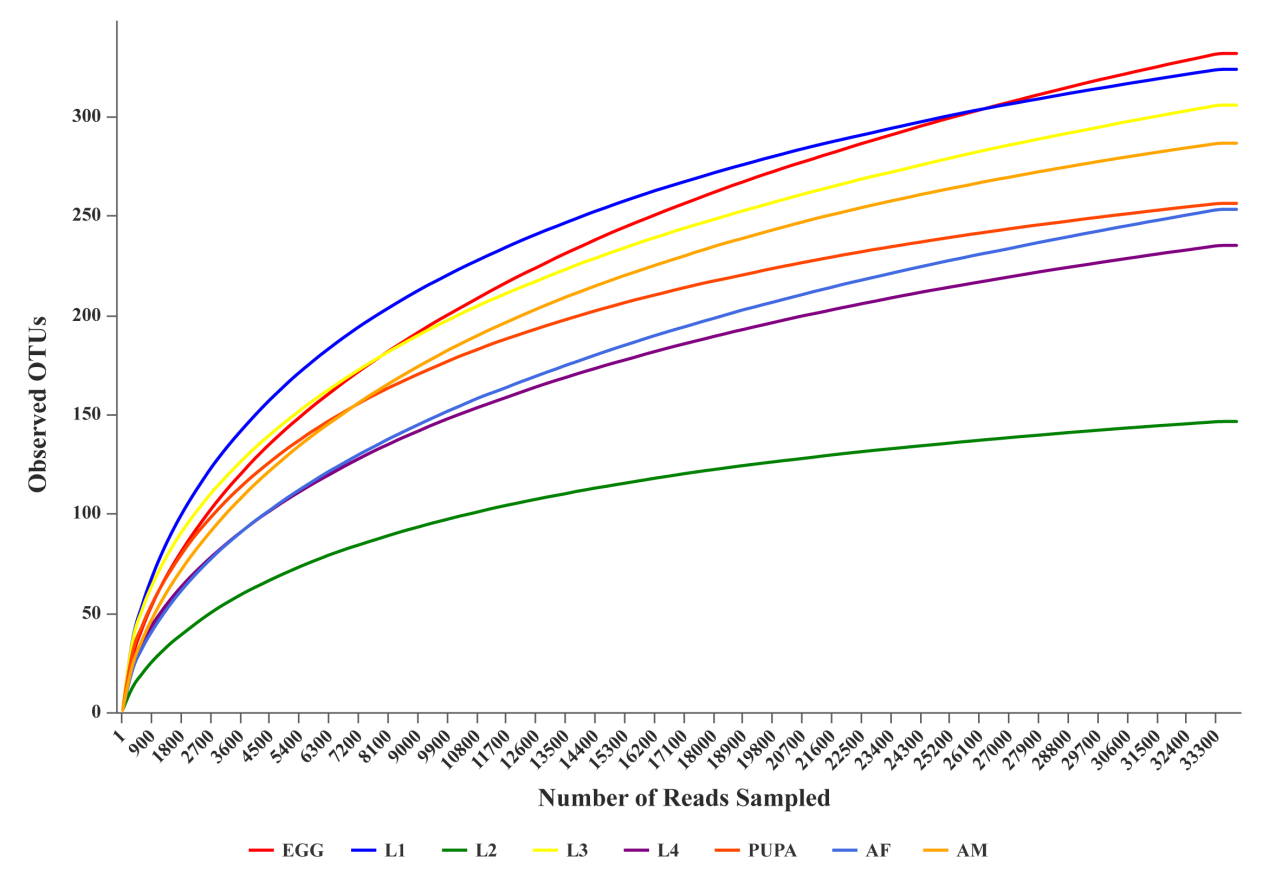


**Figure S3. Rarefaction curves of the 16S rRNA gene reads based on OTUs at 97% sequence similarity.**

##
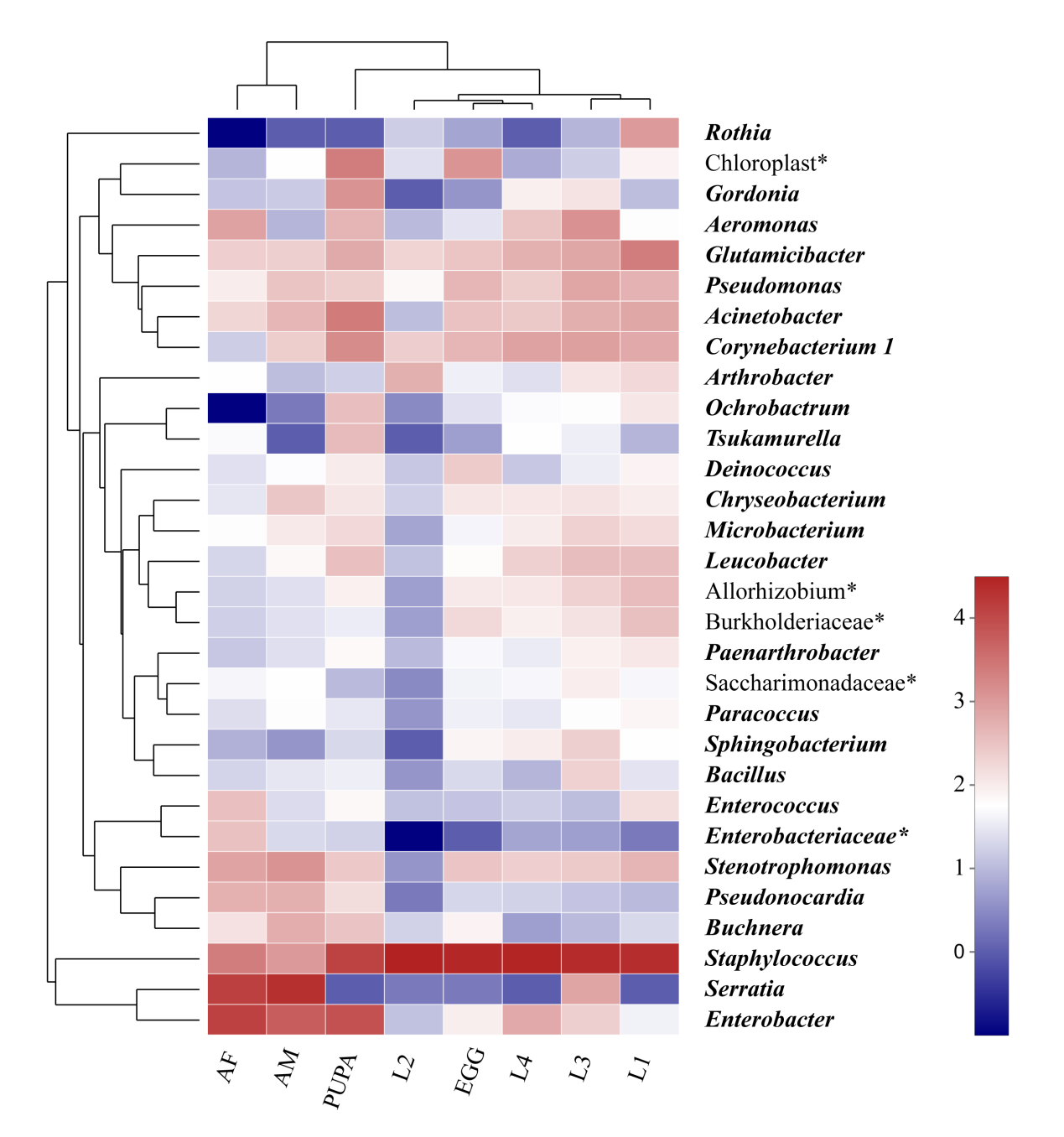


## Figure S4. Heat map of major taxa over across the H. axyridis life cycle, at the genus level. The color code indicates relative abundance, ranging from blue (low abundance) to red (high abundance). *denotes unclassified operational taxonomic units (OTU) reported at higher taxonomic level. Refer to Fig. 1 for sample abbreviations.

**
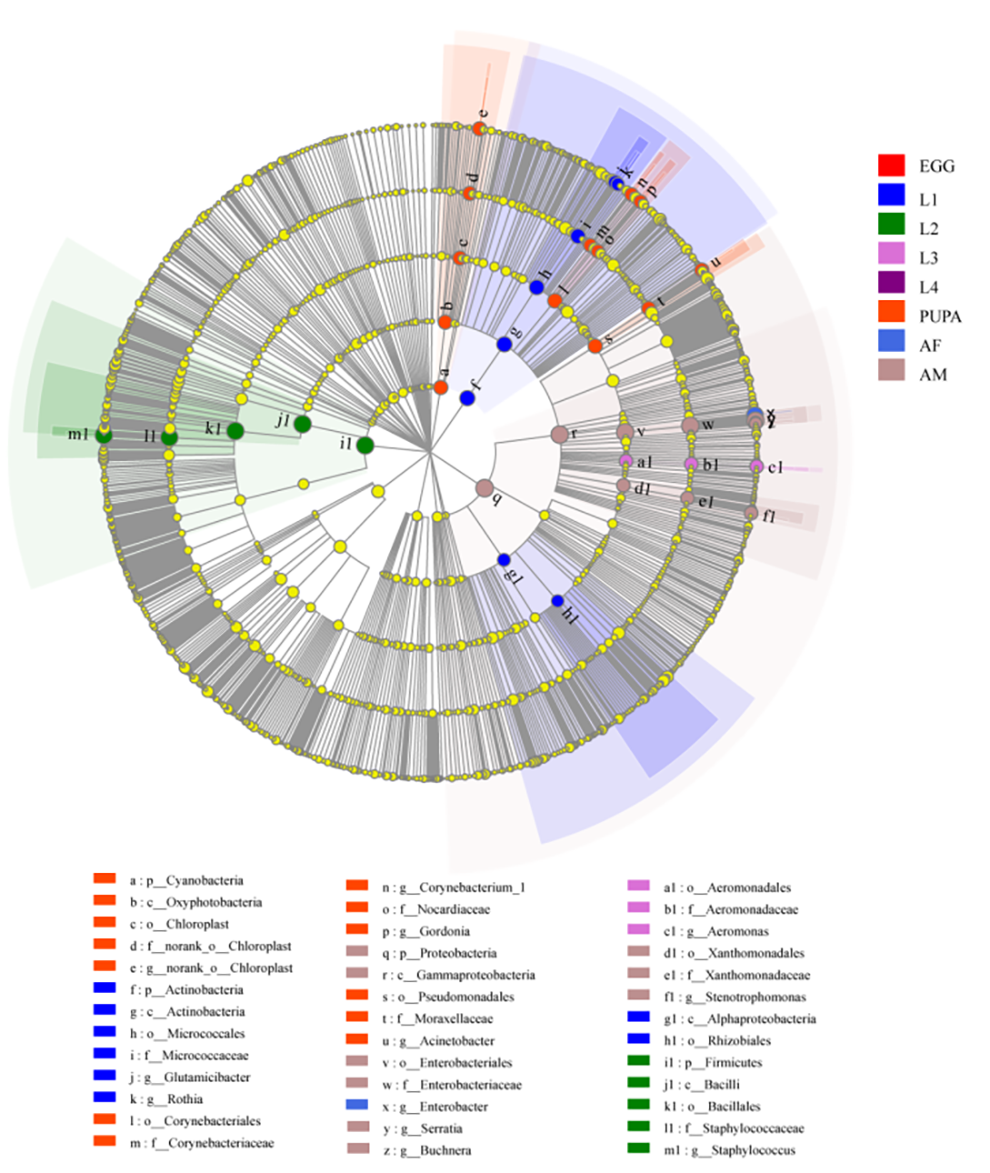
**

**Figure S5. Linear discriminant analysis effect size (LEfSe) analysis of microbial abundance across all life stages of H. axyridis. Cladogram of microbial communities.**

##
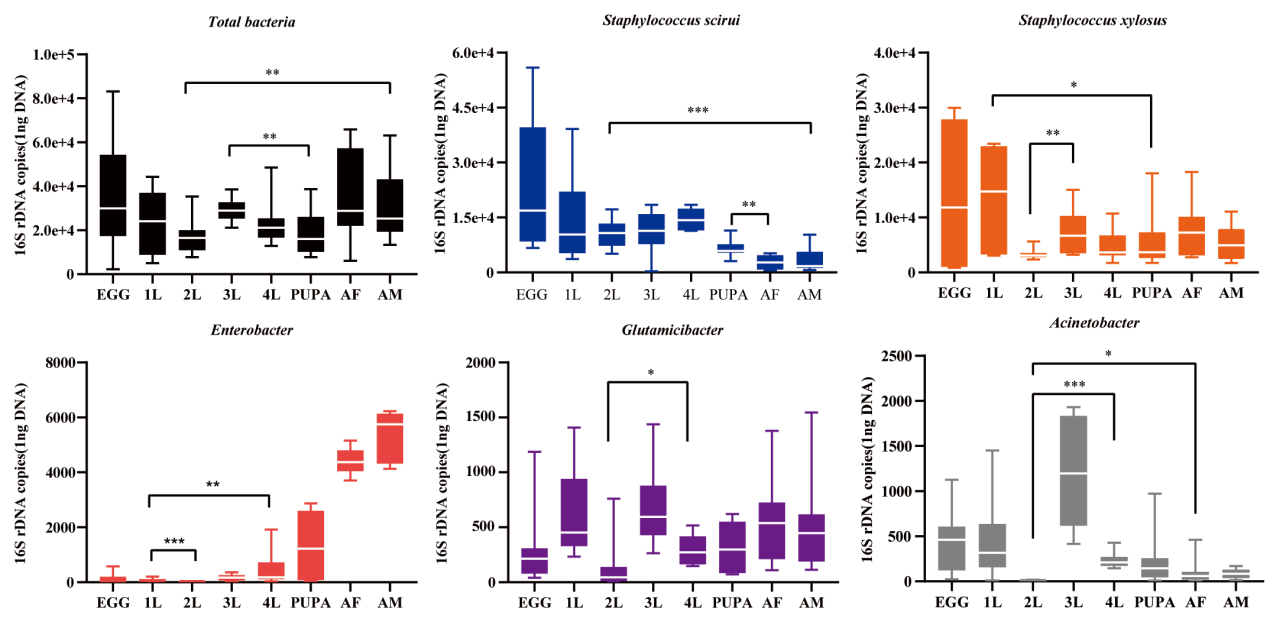


**Figure S6. Changes in gut microbiota composition across all life stages of *H. axyridis*. Shown are numbers of 16S rRNA gene copies of dominant bacterial species at different developmental stages of H. axyridis as determined by qPCR. Box-and-whisker plots show high, low, and median values, with lower and upper edges of each box denoting first and third quartiles, respectively. ** = p < 0.01 and *** = p < 0.001 (Kruskal-Wallis test).**

**Table S1 Sequencing analysis of 16S rRNA gene amplicons of *H. axyridis* with diversity indices.**

| **Samples** | **Seqs_Num** | **AvgLen (bp)** | **OTU_Num** | **Diversity indexs** | | | | |
| --- | --- | --- | --- | --- | --- | --- | --- | --- |
|  |  |  |  | **Ace** | **Chao 1** | **Shannon** | **Simpson** | **Coverage** |
| **Egg_1** | 62643 | 429 | 212 | 483.6828 | 366.8947 | 0.7334 | 0.6633 | 0.9967 |
| **Egg_2** | 62865 | 427 | 435 | 588.8123 | 543.0106 | 1.4614 | 0.4668 | 0.9957 |
| **Egg_3** | 62506 | 429 | 189 | 312.6189 | 289.7368 | 0.9799 | 0.4622 | 0.9974 |
| **Egg_4** | 65235 | 428 | 193 | 441.5304 | 340.0968 | 0.9249 | 0.5394 | 0.9971 |
| **Egg_5** | 62136 | 419 | 677 | 751.5286 | 743.1111 | 3.7403 | 0.0812 | 0.9964 |
| **Egg_6** | 59802 | 428 | 284 | 590.3654 | 417.3500 | 1.3163 | 0.4681 | 0.9962 |
| **L1_1** | 50698 | 425 | 386 | 405.2897 | 408.6579 | 2.5476 | 0.2695 | 0.9987 |
| **L1_2** | 63599 | 428 | 321 | 437.9140 | 424.2632 | 1.3190 | 0.4662 | 0.9967 |
| **L1_3** | 57452 | 428 | 257 | 383.6846 | 358.0000 | 1.0973 | 0.4631 | 0.9969 |
| **L1_4** | 59039 | 424 | 343 | 427.4806 | 423.9444 | 2.4831 | 0.2052 | 0.9972 |
| **L1_5** | 61100 | 423 | 362 | 392.2346 | 383.3871 | 2.3707 | 0.2176 | 0.9984 |
| **L1_6** | 56547 | 423 | 273 | 355.2079 | 341.6957 | 2.5329 | 0.1738 | 0.9976 |
| **L2_1** | 58834 | 429 | 149 | 160.6575 | 160.0000 | 0.7525 | 0.6722 | 0.9993 |
| **L2_2** | 60674 | 429 | 169 | 212.4666 | 211.5000 | 0.4762 | 0.8452 | 0.9985 |
| **L2_3** | 62298 | 429 | 123 | 165.3462 | 189.0000 | 0.3883 | 0.8700 | 0.9987 |
| **L2_4** | 60701 | 427 | 162 | 197.2496 | 197.0370 | 0.7813 | 0.7289 | 0.9987 |
| **L2_5** | 57401 | 428 | 196 | 220.9148 | 224.1200 | 0.7930 | 0.7721 | 0.9989 |
| **L2_6** | 60596 | 428 | 79 | 102.6737 | 98.7143 | 0.5445 | 0.7938 | 0.9993 |
| **L3_1** | 50272 | 427 | 474 | 630.9337 | 611.4675 | 2.4820 | 0.2614 | 0.9956 |

| **L3_2** | 111523 | 427 | 336 | 463.5241 | 445.0000 | 1.8419 | 0.4575 | 0.9967 |
| --- | --- | --- | --- | --- | --- | --- | --- | --- |
| **L3_3** | 125338 | 425 | 383 | 471.3639 | 458.0968 | 2.5236 | 0.2870 | 0.9971 |
| **L3_4** | 56876 | 427 | 219 | 273.5821 | 276.4412 | 1.1131 | 0.6659 | 0.9981 |
| **L3_5** | 45968 | 428 | 174 | 341.7400 | 260.3636 | 0.4211 | 0.8877 | 0.9977 |
| **L3_6** | 54324 | 425 | 248 | 308.2513 | 317.0278 | 1.8919 | 0.3952 | 0.9979 |
| **L4_1** | 51437 | 427 | 202 | 307.0714 | 279.0000 | 1.0912 | 0.6478 | 0.9977 |
| **L4_2** | 51084 | 428 | 195 | 280.1159 | 276.8485 | 0.8112 | 0.7320 | 0.9978 |
| **L4_3** | 41083 | 428 | 276 | 402.8033 | 401.6341 | 1.3094 | 0.5581 | 0.9969 |
| **L4_4** | 42376 | 427 | 299 | 347.1037 | 350.0000 | 1.5560 | 0.5233 | 0.9979 |
| **L4_5** | 53812 | 428 | 201 | 251.1970 | 254.3226 | 1.1194 | 0.6285 | 0.9983 |
| **L4_6** | 50310 | 427 | 237 | 278.2227 | 269.9038 | 0.9747 | 0.6938 | 0.9982 |
| **PUPA_1** | 55084 | 426 | 228 | 291.3623 | 289.2857 | 1.8062 | 0.3450 | 0.9980 |
| **PUPA_2** | 47615 | 425 | 299 | 347.0037 | 345.2222 | 2.6041 | 0.1535 | 0.9981 |
| **PUPA_3** | 55154 | 426 | 217 | 372.2770 | 294.2326 | 1.6568 | 0.4062 | 0.9975 |
| **PUPA_5** | 55417 | 416 | 242 | 267.4869 | 265.1538 | 2.4018 | 0.1627 | 0.9987 |
| **PUPA_4** | 52269 | 424 | 247 | 286.0323 | 290.8718 | 1.8724 | 0.2567 | 0.9982 |
| **PUPA_6** | 49618 | 426 | 304 | 320.8168 | 322.5938 | 2.1357 | 0.2689 | 0.9990 |
| **Adult(female)_1** | 34648 | 426 | 314 | 351.3814 | 356.3846 | 1.6452 | 0.5215 | 0.9983 |
| **Adult(female)_2** | 57765 | 428 | 336 | 479.3819 | 448.5000 | 1.0823 | 0.6648 | 0.9962 |
| **Adult(female)_3** | 44850 | 427 | 340 | 451.4907 | 440.4444 | 1.9388 | 0.2950 | 0.9966 |
| **Adult(female)_4** | 58366 | 428 | 285 | 429.7822 | 379.4478 | 1.7061 | 0.3130 | 0.9966 |

| **Adult(female)_5** | 48899 | 429 | 109 | 581.9582 | 231.8333 | 0.2957 | 0.9185 | 0.9980 |
| --- | --- | --- | --- | --- | --- | --- | --- | --- |
| **Adult(female)_6** | 51915 | 429 | 135 | 399.2445 | 247.5417 | 0.9669 | 0.5880 | 0.9978 |
| **Adult(male)_1** | 49242 | 429 | 122 | 293.7043 | 221.5263 | 0.7328 | 0.7188 | 0.9981 |
| **Adult(male)_2** | 49084 | 428 | 228 | 302.7184 | 288.9583 | 0.8909 | 0.6967 | 0.9977 |
| **Adult(male)_3** | 56234 | 426 | 788 | 875.5371 | 865.5063 | 2.1339 | 0.3830 | 0.9953 |
| **Adult(male)_4** | 47291 | 428 | 193 | 298.0805 | 261.2500 | 1.2140 | 0.5346 | 0.9977 |
| **Adult(male)_5** | 48812 | 426 | 342 | 399.9594 | 392.4545 | 1.8474 | 0.4594 | 0.9978 |
| **Adult(male)_6** | 51062 | 427 | 46 | 124.2032 | 70.0000 | 0.9695 | 0.6210 | 0.9995 |

**Table S2. Primers used in this study.**

| **Primer** | **Sequence, 5′-3′** | **Application** |
| --- | --- | --- |
| 338F | ACTCCTACGGGAGGCAGCA | PCR of V3 and V4 hypervariable regions of eubacterial 16S rRNA gene |
| 806R | GGACTACHVGGGTWTCTAAT |  |
| 16S-27F | AGAGTTTGATCCTGGCTCAG | qPCR of eubacterial 16S rRNA gene |
| 16S-355R | CTGCTGCCTCCCGTAGGAGT |  |
| Ss60F | GTCTTCGGATCGTAAAACTC | qPCR of *Staphylococcus sciuri* |
| Ss198R | GATAACGCTTGCCACCTA |  |
| Sx55F | TGAAGGGTTTCGGCTCG | qPCR of *Staphylococcus xylosus* |
| Sx168R | GCTGCTGGCACGTAGTTAG |  |
| Ent89F | GGAGGAAGGTGTTGAGGT | qPCR of *Enterobacter* |
| Ent221R | GCGCTTTACGCCCAGT |  |
| Glu129F | GCTAACTACGTGCCAGCAG | qPCR of *Glutamicibacter* |
| Glu247R | GGTTGAGCCTCGGACTTT |  |
| Aci87F | GAGGAGGAGGCTCTTCTAG | qPCR of *Acinetobacter* |
| Aci222R | ACGCTTTACGCCCAGT |  |

Note: F, forward; R, reverse
